# Lagging-Domain Model for Compensation of Hysteresis of xMR Sensors in Positioning Applications

**DOI:** 10.3390/s18072281

**Published:** 2018-07-14

**Authors:** Dora Domajnko, Dejan Križaj

**Affiliations:** 1 RLS Merilna tehnika d.o.o., Pod vrbami 2, 1218 Komenda, Slovenia; 2 Laboratory for Bioelectromagnetics, Faculty of Electrical Engineering, University of Ljubljana, Tržaška 25, 1000 Ljubljana, Slovenia; dejan.krizaj@fe.uni-lj.si

**Keywords:** hysteresis, magnetoresistive sensors, compensation, play operator

## Abstract

The hysteresis of magnetoresistive sensors remains a considerable cause of inaccuracy of positioning applications. The phenomena itself has been well studied and described by different physical and phenomenological models. Various biasing techniques have been proposed. However, the increased fabrication and computational price they require is undesirable. In this paper, a computational algorithm for the compensation of hysteresis of linear and rotary encoders is proposed. A lagging-domain model based on play operators is presented for prediction of hysteresis. The outlined procedure for the calibration of parameters allows the use of the algorithm for various types of encoders without knowing their exact material properties. The method was tested on different anisotropic magnetoresistive and tunneling magnetoresistive sensors. Results show that the impact of hysteresis was reduced by up to 90% without a significant increase of computational time or production costs.

## 1. Introduction

Magnetic field sensors have been constantly gaining attention [1,2] in the position and angular sensing industry due to their accuracy, reasonable price, and immunity to dirt. Aside from Hall and inductive sensors, three types of magnetoresistive sensors (xMR) are currently widely used commercially [3]. These are anisotropic magnetoresistive sensors (AMR), tunneling magnetoresistive sensors (TMR) and giant magnetoresistive sensors (GMR).

The AMR effect was discovered in 1856 by William Thompson, known also as Lord Kelvin [4]. He discovered that the resistance of iron and nickel changes if they are exposed to magnetic field. Nowadays, nickel-iron alloys, called permalloys, are used to form thin film resistors that can achieve up to 4% change in resistance in the presence of magnetic field. The effect happens due to spin-orbit interaction and s-d scattering as described in [5,6,7,8,9,10,11]. When the material is magnetized up to the saturation, the resistivity only depends on the angle between the magnetization and current density. AMR sensor has much simpler structure than TMR and GMR and was therefore the first of the three to become widely used.

The TMR [12] and GMR [13,14] effect can be observed in a similar multilayer thin film structure with two separated ferromagnetic layers. The first has its magnetization pinned by another antiferromagnetic layer. The second ferromagnetic layer can change the direction of the magnetization freely and tends to follow the external magnetic field. The resistance of the structure depends on the angle between the magnetization directions of the two layers.

xMR sensors for positioning purposes are typically used in combination with a rotating magnet or moving magnetic scale, which generate the required magnetic field [2]. The magnetization of the ferromagnetic layer within a magnetoresistive sensor aligns with external magnetic field and thus influences the sensor’s resistance. The encoder may comprise multiple xMR sensors, arranged in such a way that the relative position of the fixed sensor and the moving magnetic field source can be calculated from sensors’ resistances, measured by corresponding electronic circuit. Examples of such assemblies are described in [1,2,15,16].

The unambiguous relation between the magnetic field and magnetization orientation of the ferromagnetic layer which determines the sensor’s response is crucial for accuracy of the measurement. Unfortunately, all ferromagnetic materials are subjected to magnetic hysteresis. In high-precision applications, the error caused by hysteresis may be unacceptable, even though it can be significantly reduced by optimizing the material and technology [15,17,18,19]. Therefore, it must be removed either by design or by software compensation. Possibilities for reduction of hysteresis by design are for example various biasing techniques, described by Tumanski [15]. Biasing techniques are used to shift the working point of the sensor away from the strongest hysteresis point [18]. Furthermore, sensors can be used in a closed-loop system together with a magnetic core and compensating coil, which also improves nonlinearities and temperature drift. For AMR sensors, a flipping field-based compensation method can be used. However, all solutions by design require additional steps in manufacturing procedure and therefore increase the producing time, material costs and, in case of active biasing, power consumption.

Mathematical methods for predicting and compensating hysteresis by software could be used alternatively. For prediction of sensor’s behavior, an accurate and invertible model is needed. Various physical models for understanding the behavior of magnetic materials have been proposed. The Stoner-Wohlfarth model [20] provides good results for analyzing thin-film magnetoresistors [15], even though it is based on some severe simplifications. The Jiles-Atherton model [21,22,23] considers domain interactions as well. Its main advantage is the connection to physical parameters [24]. Energetic models for thin permalloy films such as [25] have also been proposed. However, the described physical models cannot be solved easily and are therefore more useful for understanding the phenomena rather than for implementing them as a real-time embedded system procedure. On the other hand, phenomenological models may produce similar results without simulating the complicated behavior of the material. According to [26], phenomenological hysteresis models can be divided into three groups: differential-equation-based models, such as the Duhem [27,28] and Backlash-like [29] model, operator-based models (explained in more detail in the next paragraph) and other models, for example artificial neural networks and fuzzy systems [30,31], support vector machines technique [32] or a model for the linearization of a GMR current sensor Jedlicska et al. introduced in 2008 [33,34,35].

Operator-based hysteresis models comprise a weighted integral over a set of elementary hysteresis operators [26]. The Preisach hysteresis model [36] is a numerical model that is used for the construction of various shapes of hysteresis loops with Relay operators. For position encoders, a vector upgrade [37] of the Preisach model is needed. Unfortunately, the vector Preisach model is difficult to invert and computationally complex. Additionally, the discontinuity of Relay operators may cause trouble in control systems. The Prandtl-Ishlinskii model, which was originally proposed for plastic-elasticity [27] and later used for modeling piezo-actuated stages [26], tactile sensors [38] and other [39] is a subset of the Preisach model which exploits two essential, continuous operators with a single threshold: play and stop [40]. Ordinary and generalized play and stop operators are described in detail in [41]. The play operator has been further generalized into so-called K-P operator, used by the Krasnosel’skii-Pokrovkii hysteresis model [26,40,42,43].

In this work, we propose an operator-based model for estimating the error of xMR sensors in positioning applications caused by magnetic hysteresis. It is named the Lagging-domain model due to analogy of the mathematical construction of the model to the magnetic events in the material. The use of the ordinary play operators is substantiated. A complete algorithm enabling the real-time estimation of the error due to the hysteresis is thoroughly described. Its efficiency was verified on various AMR and TMR sample sensors. At the end, the model benefits and its limitations are highlighted together with the possibilities for the future work.

## 2. The Lagging-Domain Model

**Figure 1:**
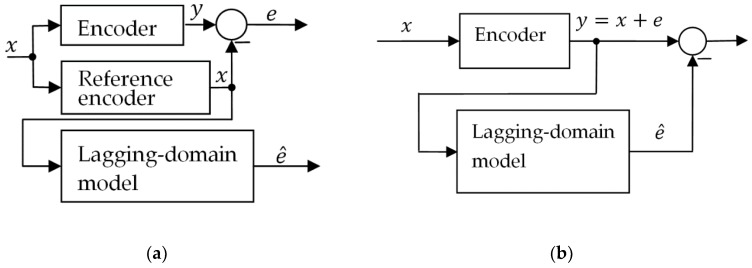
Signal diagram of the encoder and the model. (**a**) The measured error of the encoder is compared to the result of the simulation with the Lagging-domain model. (**b**) The Lagging-domain model is used for compensation of the hysteresis error of the encoder. Since the reference angle is not known in this case, the output of the encoder is used as an input of the model.

The encoder is modeled in terms of an open-loop SISO (single input, single output) system. The measured position is proportional to the direction of the external magnetic field. As the field only rotates in the *x*-*z* plane of the sensor (the setup is explained in more detail in chapter Calibration), its direction can be uniquely described by angle *θ*. In this paper, angular degrees will be used as the units for the angle of rotation for rotary or for position within a magnetic pole for linear encoders. Otherwise, they may be replaced by micrometers or other units, respectively. The model accepts the actual direction of the external magnetic field as an input and calculates the measured direction as an output. The signal diagram is shown in Figure 1a. For explanation of symbols see Table 1. The model is time-discrete as is the duty cycle of the encoder.

The idea for the Lagging-domain model comes from the magnetic behavior of the material. The external magnetic field affects magnetic domains by torque; therefore, they reform, causing energy losses. The energy provided by external field increases with the magnetic torque, the torque depends on the angle between external magnetic field and the magnetization of the domain. Some domains need more energy to turn than others. The orientation of magnetization of such parts of the material will be “dragged” behind the external field with a lag, bigger than of those which turn more easily.

Similarly, the response of an xMR sensor can be mathematically subdivided into components called domains. The domain function must be defined with respect to three requirements for the model: continuity, symmetry, and space invariance. From the operators, discussed in the first chapter, an ordinary play operator is chosen for the domain function, while the relay operator fails to provide continuity, the stop operator and the generalized play operator are not space invariant and the K-P operator is generally not symmetric.

**Figure 2:**
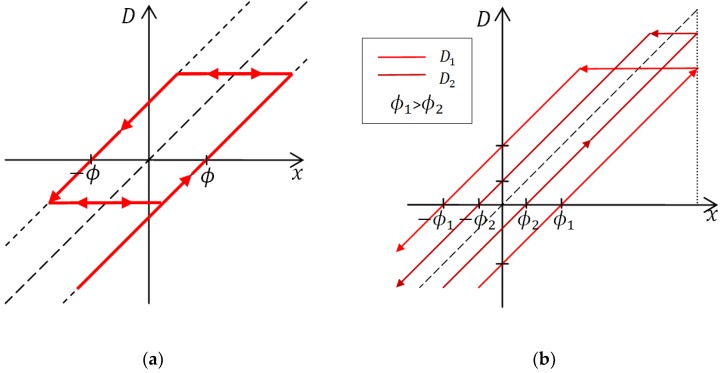
The ordinary play operator is used to describe the mathematical domain response to direction of external magnetic field *x*. (**a**) The value of the domain *D* does not change unless the difference between *x* and *D* exceeds the value of parameter *φ*. In that case, the domain response follows one of the dotted lines with a unity slope. (**b**) Responses of two different domains to the change in the direction of movement where $\phi_{1}>\phi_{2}$. The sensor was first moving upwards and then downwards along the scale.

The value of domain *D_i_* in a time step *t_j_* depends on the direction of the external magnetic field and on the history of the domain according to Equation (1), where parameter $\phi_{i}$ is the lag between directions of external magnetic field and domain magnetization in saturation. The response is represented graphically in Figure 2. Each of *N* domains has its own value $\phi_{i}$.

The hysteresis loop of the described operator-based models, including the common Prandtl-Ishlinskii model is constructed as a weighted sum of mathematical domains. The Lagging-domain model’s output is the estimated error of measurement due to hysteresis and is calculated in a slightly different way by Equation (2). This way, the output of the model can be directly used for the compensation of measurement error due to hysteresis. Also, the weights can be easily determined with the calibration routine described in the chapter *Calibration*.

(1) $$D_{i}\left(t_{j},\;x_{j}\right)=\{\begin{array}{c} x_{j}-\phi_{i}\\ x_{j}+\phi_{i}\\ D_{i}\left(t_{j-1}\right) \end{array}\begin{array}{cc} ; & x_{j}-D_{i}\left(t_{j-1}\right)>\phi_{i}\\ ; & x_{j}-D_{i}\left(t_{j-1}\right)<-\phi_{i}\\ ; & else \end{array}$$

(2) $$\hat{e}\left(t_{j},x_{j}\right)=\overset{N}{\underset{i=1}{\sum }}w_{i}\;\cdot \left(D_{i}\left(t_{j},x_{j}\right)-x_{j}\right)$$

**Figure 3:**
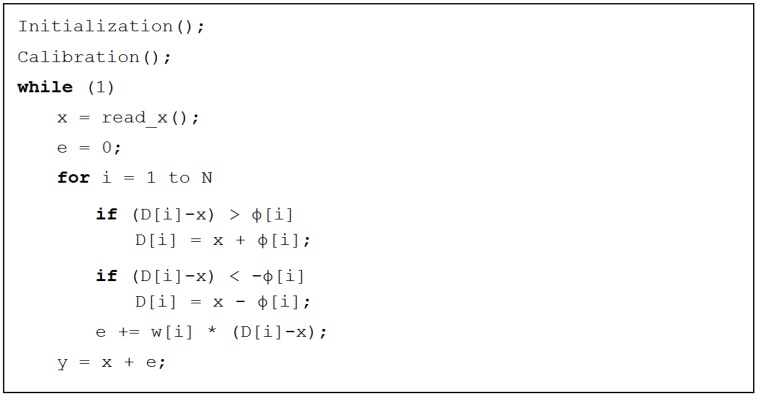
The pseudo code for modeling the sensor’s output using the Lagging-domain model. At each time step the true position of the sensor is read from the reference encoder. Then, the states of the domains are updated. The estimated error of the sensor is calculated. The estimated output of the sensor is the sum of the correct value and the estimated error.

The pseudo code of the algorithm is presented in Figure 3. We can see that the computational complexity is proportional to *N*. Computational space is taken mostly by arrays ***D***, ϕ and w which are of length *N*.

Please note that the lags *φ* do not depend on the absolute position of the encoder. The shape of the hysteresis loop only depends on the change of the position and not on the absolute point of turn. The model is therefore suitable only for modeling sensors without a significant anisotropy of the material which would result in a spatially dependent hysteresis effect. The significance of anisotropy depends on the type of the sensor and its process parameters. A quick suitability test to check the appropriateness of the sensor for the Lagging-domain algorithm can be carried out by recording the error during straight-line upward and downward movement. The difference between the two should be constant over the whole operating range.

### 2.1. Calibration

**Figure 4:**
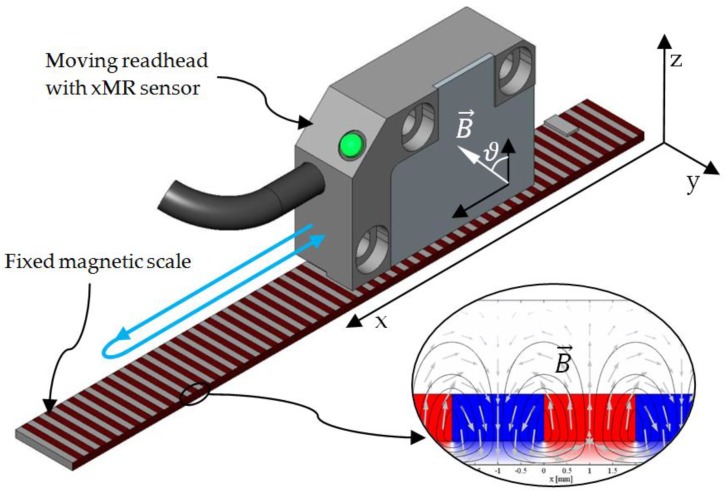
The setup of a linear encoder. A read-head with xMR sensor moves along the x axis above the magnetic scale with alternating poles, magnetized up and down along the z axis and thus providing a magnetic field in interchanging direction. The movement during the calibration is indicated with the blue line.

The shape of the hysteresis is determined by arrays ***Φ*** and ***w***. The calibration of the two is performed with the data, collected from the encoder and the reference encoder during the calibration procedure (Figure 4). The advantage of an experimental approach to calibration is that no material parameters must be known. The procedure is identical for all types of xMR sensors and their applications and can be remade anytime.

During the calibration procedure, the sensor must move monotonically in one direction and then monotonically in the reverse direction. The movement must be slow enough to exclude all transients and it must cover the range wide enough that the sensor’s delay due to hysteresis settles down to a constant value. The output of the encoder and the reference signal are collected. The measurement must be free of other sources of errors such as irregularities of the magnetic scale or higher harmonics, which often occur in position measurements. Some errors can be artificially removed after the measurement is taken as they are common to both upward and downward movement. The noise can be reduced with a non-causal filter such as a Hamming window. The algorithm will be described for the case where the output value decreases in the first part of the measurement and then increases after the change of direction. The calibration for the opposite case can be done similarly with some minor changes.

**Figure 5:**
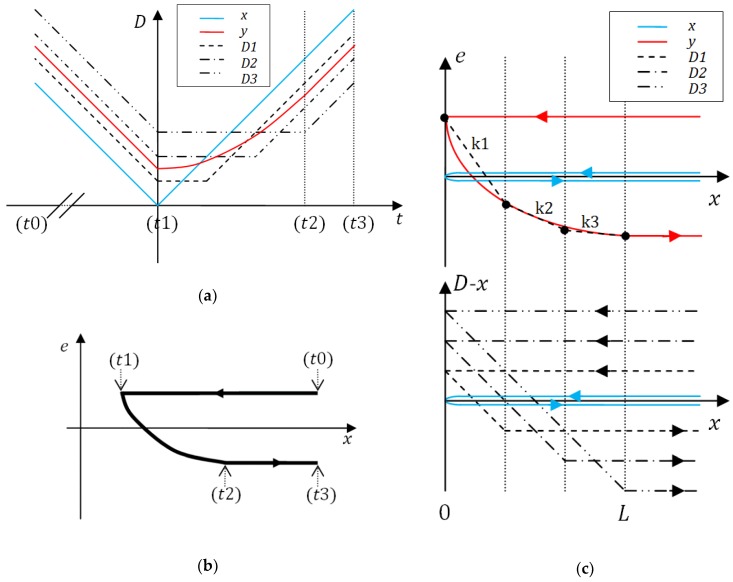
Data for calibration with identified critical points. (**a**) The collected signals *x* and *y* in time domain. The values of domains are shown for *N* = 3. (**b**) It is easier to identify critical points by observing the plot of sensor’s error *e* relative to the reference signal *x*. (**c**) The error signal *e*(*x*) between critical points (t1) and (t2) is split into *N* linear regions. Each can be expressed as a linear combination of the *N* domains.

First, four typical points are identified as shown in Figure 5. (t0) and (t3) are the beginning and the end of the measurement, (t1) is the point of turn. (t2) is the moment when the transient of hysteresis reaches saturation. The distance that the sensor makes between points (t1) and (t2) is marked with *L*. The array of values ***Φ*** is constructed by placing *N* values on the interval [0, *L*/2]. For fast implementation it is recommended to place them equidistantly in an ascending order.

The measurement error *e* is calculated by subtracting the reference signal from the sensor’s output as shown in Figure 5b. According to Equation (2), the error can be represented as a weighted sum of contributions of domains (Figure 5c). The behavior of domains can be deduced from their definition. Graph *e*(*x*) between the critical points (t1) and (t2) (shown in Figure 5b) can be split into *N* linear sections with a gradient k=Δe/Δx (Figure 5c above). By rewriting the gradients from right to left we obtain Equation (3), or, rewritten in a vector form and generalized for various number of domains, (4). The system of equations is solved by (5) to obtain the weights.

(3) $$\begin{array}{c} -k_{3}=w_{3}\\ -k_{2}=w_{2}+w_{3}\\ -k_{1}=w_{1}+w_{2}+w_{3} \end{array}$$

(4) $$-k=\left[\begin{array}{cccc} 1 & 1 & \cdots & 1\\ 0 & 1 & \cdots & 1\\ \vdots & \vdots & \ddots & \vdots \\ 0 & 0 & \cdots & 1 \end{array}\right]*w$$

(5) $$w=-{\left[\begin{array}{cccc} 1 & 1 & \cdots & 1\\ 0 & 1 & \cdots & 1\\ \vdots & \vdots & \ddots & \vdots \\ 0 & 0 & \cdots & 1 \end{array}\right]}^{-1}*k$$

In general, vector ***Φ*** does not have to depend on the calibration procedure but can be chosen in advance. In this case, *L* must be bigger than the expected range of a hysteresis effect. However, the accuracy of the model can be optimized by varying combinations of *N* and *L*.

### 2.2. Compensation

The Lagging-domain model is used to estimate the current error of the xMR encoder. Once the error is known, it can be subtracted from the output angle and thus eliminated. Since the reference signal *x*, which was the input of the model, is not known, the output of the sensor is used instead (see Figure 1). There is a slight mismatch between the two, but its impact can be reduced by using the sensor’s output instead of the reference signal already in the calibration routine. The compensation algorithm may be built in the encoder’s microprocessor or added as a post-processing module.

## 3. Experiment and Results

**Figure 6:**
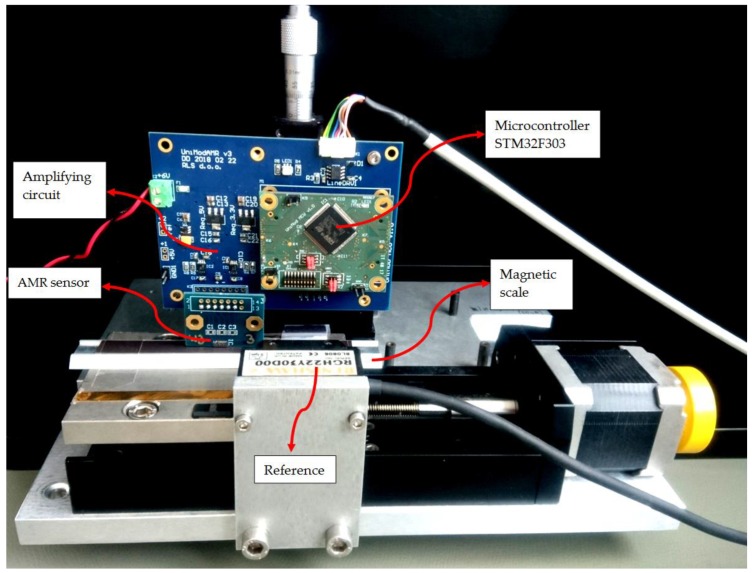
The setup for the measurements. The xMR sensor is attached to an electronic circuit, which provides power supply, signal amplification, A/D conversion, a signal processing unit, and a communication unit for data transmission. Beneath the sensor is the moving magnetic scale. A very accurate reference encoder is needed for calibration and verification.

The developed algorithm was tested on AMR and TMR linear sensor samples. The magnetic field of a sinusoidal shape was provided by a magnetic scale with alternating, 2  mm long magnetic poles. Sensing elements were connected in a double Wheatstone bridge and were thus providing the information about the sine and cosine component of magnetic field. The setup for testing is shown in Figure 6. A linear stage with an optical reference encoder with the resolution of 0.1 µm, which exhibits no significant hysteresis, was used for accurate measurements of the linear position. The magnetic scale was placed right next to the scale for the optical encoder so that there were no moving mechanical parts between them. Therefore, there were no additional hysteresis effects caused by the reference encoder or the stage which could be unwittingly mistaken for the error of the MR encoder. The signal acquisition and processing was performed partly by the microcontroller and partly by PC with MATLAB.

The data from sensing elements were collected along with the reference position. After some basic calibration of the signals, the angle of magnetic field above the magnetic scale was calculated from the sine and cosine signal. The position along the horizontal axis is proportional to this angle. The distance of 360° corresponds to one electrical period of the signal, which is one magnetic pole for AMR and one magnetic period (two poles) for TMR sensor.

In multi-turn applications with single-turn encoders, the overflow between 360° and 0° must be considered. The proposed solution is that whenever an overflow of the input signal is detected, all domains are accordingly transferred for 360°. This may sometimes result in an output which is not within the [0°, 360°) interval, but this can be corrected by division by module 360.

**Figure 7:**
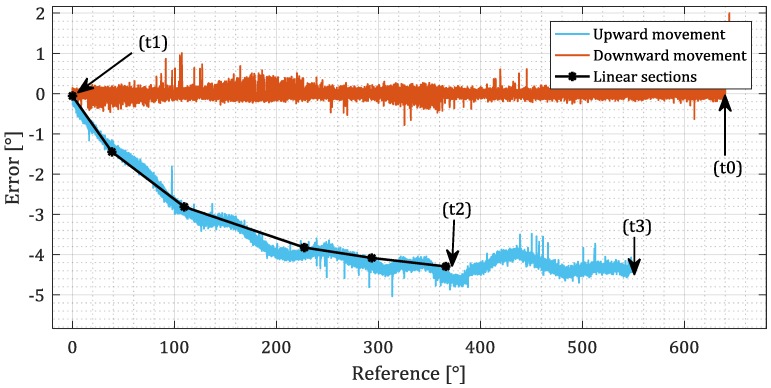
Data collected for the calibration-the error of the encoder during downward movement, a turn and then the upward movement. The typical points are identified. The section between (t1) and (t2) is split into five linear regions.

Before the first use, the calibration sequence was carried out. The sensor was moved as proposed in the chapter *Calibration*. The collected data are shown in Figure 7. The number of domains *N* was set to 5. Vector ***Φ*** was determined. The data were split into linear regions and vector ***w*** was calculated according to Equation (5). Then, vector ***D*** was initialized. Since no information about the state of the material before the beginning of simulation is available, a demagnetized state is assumed, and ***D*** was filled with random numbers from range [0°, 360°).

**Figure 8:**
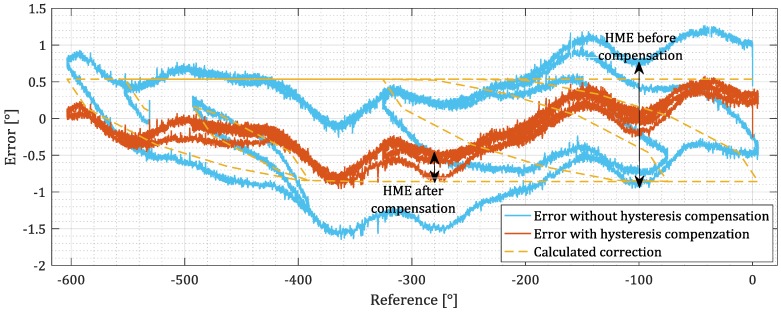
Error of the sensor with and without hysteresis compensation. Before the compensation, a noticeable gap between the error during upward and downward movement can be seen. The gap is decreased after the compensation is applied. The remaining error is mostly due to the imperfections of the magnetic scale.

After initialization and calibration of the Lagging-domain model of a sensor, the measurements were taken. The sensor was moved in an arbitrary sequence. The reference signal and the output of the sensor were collected. The actual error was calculated by subtracting the reference signal *x* from the output *y* (Figure 8, blue). Then, the Lagging-domain model was used to calculate the estimated error, using the reference signal as the input (Figure 8, dotted). The estimated error was used to compensate the effect of the hysteresis at the output of the sensor by subtracting it. The difference between the measured value after correction and the reference is shown red in Figure 8.

For the validation of the compensating algorithm, the test for quantitatively evaluating the error due to hysteresis had to be defined. The sensor was moved over the whole operating range with occasional changes of direction. The severity of the hysteresis impact on the measurement error (HME) was evaluated by finding the maximal difference of a measurement at the same position during upward and during downward movement. HME is expressed in the same unit as the error, in our case angular degrees. Please note that the noise may also false contribute to the HME.

**Figure 9:**
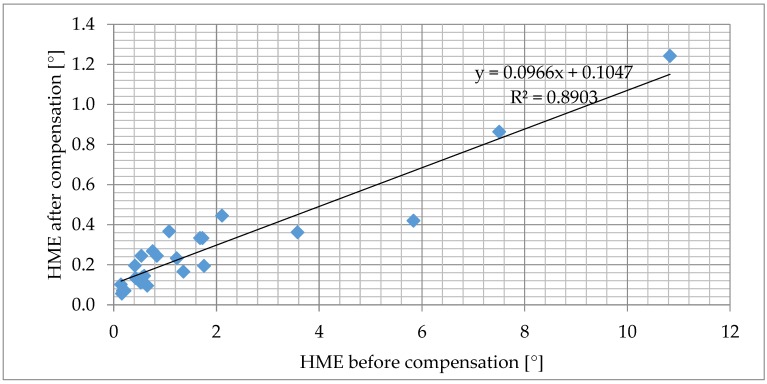
The efficiency of the compensation algorithm was tested on 10 different types of AMR sensors by comparing the indicator HME before and after compensation. The hysteresis before the compensation depends mostly on the sensing distance and on the method of construction.

**Figure 10:**
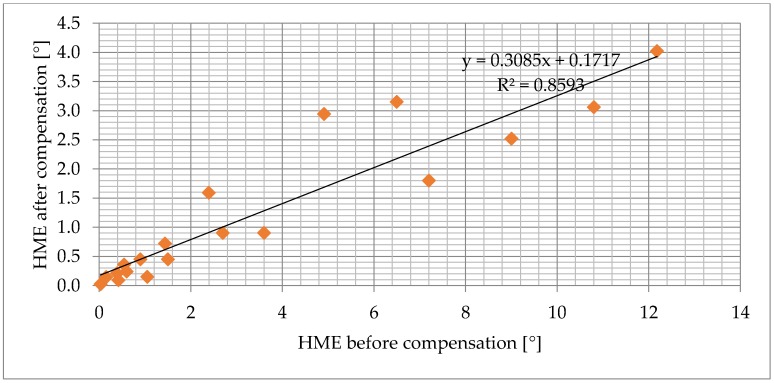
The efficiency of the compensation algorithm, tested on 6 different TMR sensors. The algorithm performs worse on TMR than on AMR sensors. The result is expected because some of the TMR samples hardly fulfill the test for suitability for use with the Lagging-domain algorithm.

The efficiency of the compensation was evaluated by comparing the HME before and after the compensation as shown in Figure 8. The result of tests performed on AMR sensor samples is shown in Figure 9. The result for tests on TMR sensors is in Figure 10. The detailed experimental data and samples descriptions are provided in the Appendix A. The tests have shown that the impact of hysteresis was reduced by up to 90% for AMR and by 70% for TMR samples.

The Lagging-domain algorithm with 5 domains was implemented into an encoder with the STM32F303 microcontroller. The duty cycle was extended for approximately 5 µs. Refresh rates of similar encoders are typically 20 to 50 µs. The latency, caused by described algorithm, is therefore just within the acceptable range. The power consumption was not noticeably affected.

## 4. Discussion

The algorithm for compensating the error of xMR motion sensors due to the hysteresis effect using the Lagging-domain model was developed and tested on different AMR and TMR sensor samples. The resemblance of the concept of xMR sensors suggests that the model may be suitable for all three types. However, there may be exceptions, so the properties of material must be checked by performing the suitability test before applying the algorithm.

The algorithm cannot calculate the error prediction correctly directly after the start-up. The state of the material before the turn-on of the encoder is unknown and the domains are therefore initialized to an arbitrary state. The encoder must move far enough to align the domains in the right position before the model converges to the right value. We suggest moving a sensor for at least one magnetic period after the start-up before the first use. However, a similar movement is required for all quasi-absolute positioning systems with an incremental scale and a reference mark, which must be reached to set the zero position. Because xMR sensors are often used in such systems, the compensating algorithm may be set up during the scan for the reference.

The algorithm does not include any temperature compensation. The hysteresis effect in general depends on the temperature because it affects the movement of the magnetic domains in the material. However, in applications that need the accuracy in the range of noticeable hysteresis, the temperature must be kept stable due to thermal expansions. Therefore, there is no need for additional temperature monitoring.

The proposed Lagging-domain model is designed for the encoders where the sensing distance does not change after the installation and calibration. In the future work, the extension for variable magnitude of magnetic field may be considered. The hysteresis increases significantly with the sensing distance. The solution may lie with the concurrent adaptation of parameters *φ*.

The computation time of the described algorithm is within the acceptable range. However, the speed of the algorithm could be further improved. The number of operations is proportional to the number of domains. Shorter computation time could be achieved by optimizing the code or by using fewer domains.

## 5. Conclusions

An algorithm for the compensation of hysteresis error for the linear and rotary encoders using the Lagging-domain model is proposed. The play operators were used for modeling the magnetoresistive sensors in a positioning application for the first time. The tests have shown that the Lagging-domain algorithm reduces the hysteresis of AMR encoders by up to 90%. The implementation into an embedded system with a microcontroller requires no additional resources. The duty cycle of the encoder was extended for approximately 5 µs. The calibration of the system can be performed within a microcontroller or externally, as long as the required moving sequence can be performed.

## Figures and Tables

**Table 1:** List of used symbols.

Symbol	Meaning	Unit
N	Number of mathematical domains	/
D	Domain function-play operator	°
$\vec{B}$	Magnetic flux density	mT
$\phi =\left\{\phi_{1},\;\phi_{2},\;\ldots ,\;\phi_{N}\right\}$	Domain lags-parameters for play operators	°
$D=\left\{D_{1},\;D_{2},\;\ldots ,\;D_{N}\right\}$	Current domain orientation angles	°
$w=\left\{w_{1},\;w_{2},\;\ldots ,\;w_{N}\right\}$	Weights	/
x	Actual angle of $\vec{B}$ in *x*-*y* plane, measured by a reference encoder	°
y	Measured angle of $\vec{B}$; encoder’s output, affected by hysteresis	°
e=y−x	Error of measured angle	°
$\hat{e}$	Estimated error	°
t	Time	s
$t_{i}$	Time step	/

## Appendix A

Full experimental data is provided. All experiments on AMR sensor samples are described in Table A1 and all on TMR samples in Table A2. Experiments were conducted for various starting HME values, which depend mostly on the sensing distance and the sensor quality.

**Table A1:** The experimental data for AMR sensor samples.

Product Name/Mark	Company	Electrical Period [μm]	HME Before Compensation [°]	HME After Compensation [°]	HME Before Compensation [μm]	HME After Compensation [μm]	Relative Improvement
5B	Under NDA ^1^	2000	1.727	0.333	9.594	1.848	81%
		2000	1.680	0.333	9.332	1.852	80%
		2000	7.507	0.864	41.708	4.800	88%
		2000	0.427	0.133	2.373	0.738	69%
A5	Under NDA	2000	0.540	0.244	3.001	1.358	55%
SM2L [44] #1	iC Haus	2000	0.761	0.268	4.226	1.489	65%
		2000	2.109	0.445	11.714	2.471	79%
		2000	0.416	0.196	2.314	1.087	53%
		2000	1.081	0.366	6.007	2.035	66%
W3_2	Under NDA	2000	1.359	0.166	7.550	0.921	88%
		2000	0.159	0.056	0.881	0.310	65%
W3_1	Under NDA	2000	0.552	0.137	3.067	0.760	75%
		2000	0.178	0.070	0.987	0.389	61%
W5_8	Under NDA	2000	0.653	0.096	3.625	0.533	85%
		2000	1.225	0.232	6.806	1.290	81%
		2000	0.217	0.070	1.207	0.388	68%
W5_3	Under NDA	2000	0.595	0.145	3.306	0.805	76%
		2000	3.578	0.362	19.876	2.014	90%
7A	Under NDA	2000	1.759	0.194	9.770	1.077	89%
		2000	0.531	0.111	2.952	0.614	79%
2C	Under NDA	2000	10.829	1.242	60.163	6.902	89%
		2000	5.835	0.420	32.418	2.334	93%
SM2L [44] #2	iC Haus	2000	0.840	0.245	4.669	1.358	71%
		2000	0.144	0.101	0.799	0.561	30%

^1^: Under a non-disclosure agreement. Only an internal experimental labeling of the sample is provided.

**Table A2:** The experimental data for TMR sensor samples.

Product Name/Mark	Company	Electrical Period [μm]	HME Before Compensation [°]	HME After Compensation [°]	HME Before Compensation [μm]	HME After Compensation [μm]	Relative Improvement
TL912 z [45]	Sensitec	1000	6.497	3.152	18.046	8.755	51%
		1000	4.908	2.940	13.634	8.167	40%
		1000	12.179	4.022	33.830	11.172	67%
		1000	0.548	0.358	1.524	0.996	35%
		1000	2.392	1.589	6.644	4.413	34%
		1000	0.155	0.146	0.430	0.406	6%
MDT TMR40xx package [46]	Multi Dimension Technology (MDT)	1200	0.030	0.021	0.100	0.070	30%
MDT TMR40xx custom design	Multi Dimension Technology (MDT)	1200	1.050	0.150	3.500	0.500	86%
		1200	0.420	0.090	1.400	0.300	79%
		1200	0.600	0.240	2.000	0.800	60%
		1200	0.390	0.210	1.300	0.700	46%
TMR4012 [47]	Multi Dimension Technology (MDT)	1200	1.500	0.450	5.000	1.500	70%
MIS6301 [48]	Multi Dimension Technology (MDT)	2000	1.440	0.720	8.000	4.000	50%
		2000	0.900	0.450	5.000	2.500	50%
		2000	3.600	0.900	20.000	5.000	75%
		2000	2.700	0.900	15.000	5.000	67%
		2000	10.800	3.060	60.000	17.000	72%
TL912 [45] c	Sensitec	1000	9.000	2.520	25.000	7.000	72%
		1000	7.200	1.800	20.000	5.000	75%
